# Interactive Timeline Approach for Contextual Spatio-Temporal ECT Data Investigation

**DOI:** 10.3390/s20174793

**Published:** 2020-08-25

**Authors:** Andrzej Romanowski, Zbigniew Chaniecki, Aleksandra Koralczyk, Mikołaj Woźniak, Adam Nowak, Przemysław Kucharski, Tomasz Jaworski, Maja Malaya, Paweł Rózga, Krzysztof Grudzień

**Affiliations:** 1 Institute of Applied Computer Science, Lodz University of Technology, Stefanowskiego 18/22 str, 90-924 Łódź, Poland; zch@kis.p.lodz.pl (Z.C.); 210911@edu.p.lodz.pl (A.K.); mpwozniak@ubicomp.pl (M.W.); 203151@edu.p.lodz.pl (A.N.); pkuchars@kis.p.lodz.pl (P.K.); tjaworski@iis.p.lodz.pl (T.J.); 217723@edu.p.lodz.pl (M.M.); kgrudzi@kis.p.lodz.pl (K.G.); 2 Institute of Electrical Power Engineering, Lodz University of Technology, Stefanowskiego 18/22 str, 90-924 Łódź, Poland; pawel.rozga@p.lodz.pl

**Keywords:** data analysis, visualization, flow investigation tool, ECT, spatio-temporal data interpretation

## Abstract

This paper presents a novel approach to a complex process of electrical capacitance tomography (ECT) measurement data analysis. ECT is frequently employed for non-invasive monitoring of industrial process phenomena. Proposed methodology is based on the premeditated integration of the spatial and temporal relations inherent in the measurement records into the workflow of the analysis procedure. We propose a concept of interactive timeline that enables arranging data visualization according to the user’s current focus along the process of analysis. We evaluated the proposed method using a prototype system in a task-based user study conducted with a group of domain experts. The evaluation is based on gravitational silo flow measurement datasets. Proposed prototype system enables diverse data manipulation in a more natural way allowing the user to switch back and forth between space and time domains along the data analysis trail. Experiments with the prototype system showed that the accuracy and completion times have significantly improved in comparison to the performance measured in the baseline condition. Additionally, the participants reported decreased physical load with improved efficiency measured with NASA task load index. Finally, a short discussion coupled with directions for the future of interactive spatio-temporal ECT measurement data analysis conclude the paper.

## 1. Introduction

Industrial process tomography systems belong to a special class of non-invasive and non-intrusive sensing systems. The most prominent feature of tomography system is the capability to measure and visualize the interior of industrial reactors and installations, the ones constructed with the use of opaque materials [1,2,3]. There have been reports on the use of electrical tomography systems for control purposes of various industrial processes, since they provide a reliable stream of quality measurement data [4,5,6]. Electrical capacitance tomography (ECT) has proven to be fast and efficient, particularly for processes that occur in closed environments [3,7,8]. Current development of ECT systems is focused on increasing the accuracy and sensitivity of measurement units [9] as well as efficiency and precision of algorithms for image reconstruction and data processing [10,11,12], which are the crucial elements for deep analysis and advanced process control [13,14,15,16,17]. Yet, prior to incorporating ECT into the process control systems, it is necessary to develop a data processing algorithm dedicated to a particular application. Therefore, it is vital to comprehensively understand the data and its connection to the real phenomena in any particular case [18]. Only the correct data processing and accurate analysis of measurement signal changes will enable the appropriate response of the control system [19].

Besides the ultimate goal of using process tomography technology as an element of the control system, its secondary goal is to investigate the processes as ECT is well adapted for it. It is a perfect tool for understanding the phenomena and changes taking place within the ongoing flows [1,2,20]. Correct interpretation of the measurement data has to be conducted by fully covering phenomena occurring in time and space domains since it is crucial for understanding the behavior of industrial processes [21,22]. Presently experts play an indispensable role in interpreting the data, especially that there are deficiencies in the automation of tomography measurement data analysis [23,24]. The complexity of both the process to be controlled as well as the measurement procedure itself makes the data extremely difficult to read and process in a generalized, universal manner [25].

Hence, there is a critical need to provide efficient paradigms for the development of the appropriate software tools for visualizing, browsing and manipulating measurement records’ datasets that enhance the current capacity of the widely used, domain-standard data analysis software packages such as MATLAB [7,26,27]. Novel methods of data analysis, such as employing large multi-touch display surfaces for operating visuals coupled with proper data presentation methods have been reported to increase the convenience as well as efficiency of similar procedures in other areas [28,29,30,31,32]. Dedicated, tangible interfaces enable users to intuitively navigate through the reconstructed images, graphs or tomograms, enabling the measurements to be explored easily in the time and space domains [33]. Such possible arrangements are of vital importance, as they not only reduce human expert workload and effort, but may also contribute to the development of improved strategies for future process control, which is unachievable by the classical manual analysis.

Moreover, experts currently have little support in the form of dedicated high-quality tomography measurement data analysis and visualization solutions. In particular, the inherent relations between the time stamps (conveyed in sequences of measurement data frames) for the ongoing process and the analyzed spatial features (visible in reconstructed 2D and 3D images) are extremely important are extremely important in the aspect of interpretation. And yet, so far these are the most difficult features to be translated into convenient software analysis and manipulation tools [34]. Applying proper visual forms for depicting process tomography data can improve performance significantly, contributing to better understanding of the examined process [35,36]. It is particularly problematic to clearly present the interdependent 2D time series visuals interconnected with various types reconstructed images of dynamic, volumetric phenomena. The main difficulty lies in maintaining the time coherency of the recorded data as perceived by the human operator analyzing the particular process [11]. Hence, it is important to develop methods for proper visualization that preserve time and space relations explicitly.

The main contribution of this work is a novel method of working with process tomography measurement data aiming at disclosing specific important information relevant to the context of the industrial process state. The proposed method is based on incorporation of the specifics of the measurement data consisting of close, spatio-temporal relations, inherent in the monitored process and preserved by the measurement tool. We show a method of contextual data analysis that arranges data visuals around the current user focus by constantly adjusting the adjacent data following along user analysis’ workflow. This novel method is based on the concept of the interactive timeline layer, which organizes the data composition in compliance with the specific purpose of the ongoing analysis. We incorporated the spatio-temporal relations into the core of the proposed method to increase effectiveness of analysis. The proposed interactive mechanism for contextual switching between time and space domains has been carefully examined in controlled laboratory settings. The approach was verified in a task-based user study and the evaluation was conducted using ECT data for the gravitational flow of bulk solids in silos [34]. Evaluation of the proposed approach showed improved performance in terms of both the efficiency and accuracy, while also decreasing the users’ workload. Another advantage reported by the users is that it enables much easier analysis and interpretation of the data in case of investigation of the general behavior of the industrial process. It is worth emphasizing that although ECT tomography measurement modality coupled the experimental data of gravitational flow of solids, used in this evaluation, the entire approach is easily applicable to the entire class of fast process tomography systems. Proposed methodology is generalizable for the electrical tomography systems, fast X-ray systems and the similar ones in the circumstance that the interpenetration of information between the space and time domains is of significant importance.

The rest of the paper is arranged as follows. Section 2 provides background on the complexity and properties of process tomography ECT measurement data as well as the insights into the current analytical procedures. Section 3 introduces the proposed interactive spatio-temporal contextual data analysis approach and gives insight into the prototype system that has been built to evaluate the proposed concepts. Section 4 presents the assumptions and hypotheses about verification of the proposed concept as well as user study design and evaluation methodology. Additionally, experimental conditions and set-up is included, along with the detailed description of the industrial process of silo flow coupled with general and specific meaning and interpretation of ECT data for this exemplary process. Then, Section 5 presents quantitative (timing measures, accuracy) and qualitative (task load index values and additional findings) results of user study decoded from experiments as well as presents interpretation and discussion of the outcomes. This section is summarized with reflection on the evaluation of hypotheses, limitations and directions for future work. The paper is concluded in Section 6.

## 2. Specificity of ECT Measurement Data

Process tomography provides numerous measurement and exploration tools, facilitating the online monitoring and diagnostics of industrial processes in a non-invasive manner. In addition to that, the captured data enable further, extensive analysis of the process, which reveals the causes of possible disturbances and provides functional parameters. These outcomes may eventually lead to better design of process facilities as well as improved control systems. [21]. The main advantage of process tomography systems compared to other measurement techniques is the ability to offer visualization of the process coupled with tracking the changes taking place in both the space and time domains on the go [26]. This section provides more details into the specifics of the ECT data, i.e., interconnected formats and volumes as well as a description of how the analytical procedures are conducted currently. But first, in order to provide readers with a better understanding of data analysis challenges, a short explanation of the silo flow is presented. We discuss an industrial process chosen for the experimental data source within this empirical research work. In conclusion of this section authors’ claimed contributions are outlined, preceded by meaningful findings from the related work and a short summary of claimed contributions.

### 2.1. Spatio-Temporal Aspects of ECT-Monitored Process Measurement Data

The main goal of this work is to explore spatio-temporal relations inherent in the tomography measurement data. Consequently, we chose an ordinary, industry-widespread, representative process of bulk solid flow as an exemplary case of ECT-monitored industrial flow. In particular, we examine the procedure of analysis and interpretation of gravitational bulk solid silo flow as an instance of the entire class of easily generalizable industrial measurement problems.

**Figure 1:**
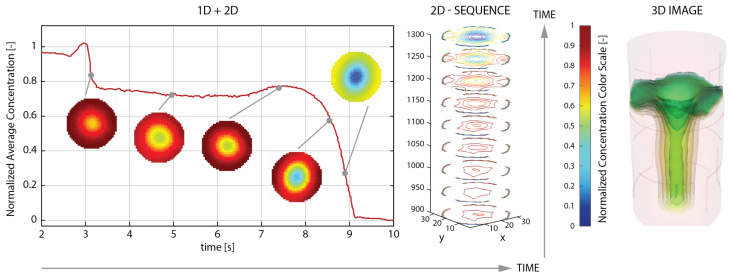
ECT data visualizations. (**Left**) 1D average concentration time plot combined with 2D reconstructed images for selected measurement frames showing the development of funnel flow over time; (**Middle**) sequence of 2D images formed in a vertical time stack; (**Right**) a single 3D reconstructed image of the silo flow with a flowing funnel feature visible.

The full set of data, received in the form of a temporal sequence of raw measurement data as well as spatial 2D or 3D images, provides a basis for process analysis. Figure 1 illustrates basic types of ECT data visualizations: from 1D averaged-concentration-values time plot coupled with 2D time-point-reconstructed cross-sectional images, to a stack of 2D segmented-images time sequence, to a single 3D reconstructed image of the single time-point volumetric process state.

**Figure 2:**
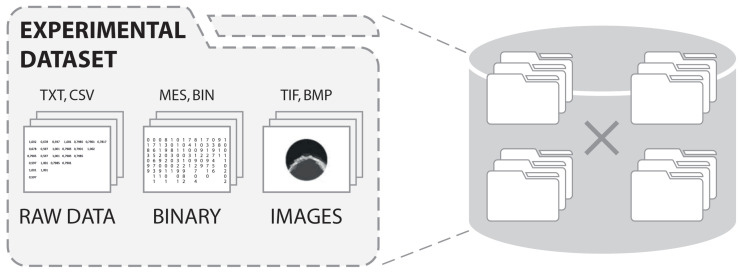
Process tomography measurement data: illustration of a typical experimental data set consisting of a variety of data types and formats (**left**) aggregated into a larger data collection repository (**right**).

Process tomography measurement datasets can contain a substantial volume of files, which first must be saved on a disc and later analyzed in an effective way [37]. Figure 2 shows an example of ECT experimental data representation. The single experiment of approximate duration of 280 s recorded at 50 fps for an eight-electrode sensor/single plane results in 14,000 measurement frames with a dataset package of approximately 462 MB for text and binary files with 2D images. This number doubles up to 924 MB for a twin plane sensor and to 934 MB and 948 MB for 12- and 16-electrode sensors, respectively.

When it comes to storing and processing 3D reconstructed imagery, the numbers again rise substantially. Each 3D image for a measurement frame captured with a 32-electrode sensor is at least 5 MB in size (or more depending on the density of the 3D reconstruction mesh that controls the output resolution). This gives an additional output of approximately 5 GB for every 1000 measurement frames. For nonlinear, iterative image reconstruction algorithms (where the sensitivity matrix is updated for each iteration of the reconstruction), up to more than 100 intermediate stages may be stored for each frame. When 1 GB of 3D data is recorded for each measurement frame, (for both the sensitivity matrix and the resulting intermediate image), this gives around 1 TB for every 1000 frames. Specific properties of the ECT process tomography data make it necessary for it to be analyzed in a broader context than just the context of the isolated measurement records. Understanding the process based on one experimental dataset requires analysis of the other datasets as well.

### 2.2. Current Analytical Procedures

Finding the key changes in measured values, over both space and time, and correlating them with a change in the state of the monitored process is the main challenge for researchers involved in the development of flow control systems [15]. The current baseline for addressing this challenge is to involve a specialist equipped with a dedicated tool for browsing and exploring complex data structures collected from the measurement system [1,2]. Then, the specialist can systematically examine tomographic measurement data using mostly self-developed, specific procedures devoted to a particular process and particular tomography modality. The correlation between distinct types of data visualization including discrete signal changes in the space and time domains is the most significant aspect of the proper data interpretation and process understanding. Then it is necessary to synthesize and transfer the products of the analysis and interpretation into a set of meaningful parameters [15].

**Figure 3:**
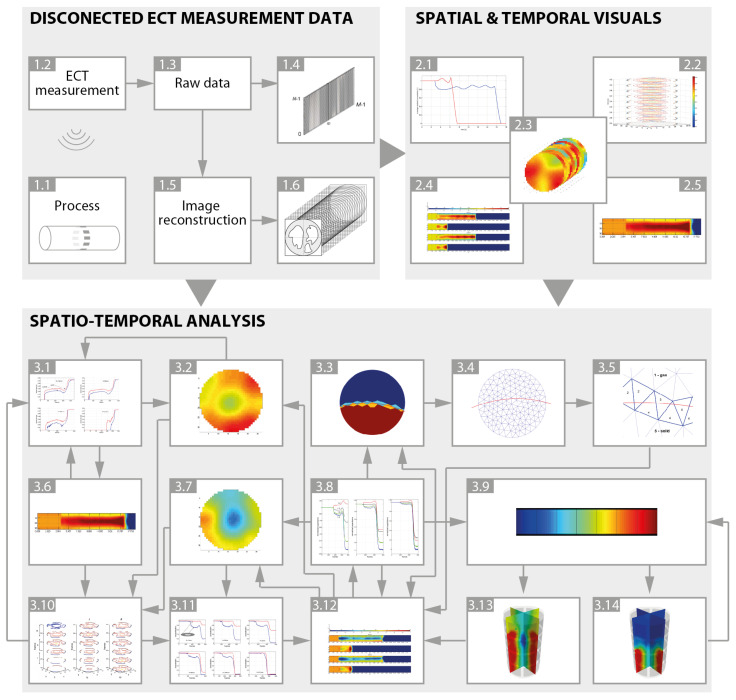
Three levels of measurement data analysis. Block #1 (**upper left corner**) illustrates the capture and preparation of measurement records in the form of raw data vectors and initially reconstructed image streams. Block #2 (**upper right corner**) represents carefully generated visuals of temporal sequences of values and 2D images. Block #3 (**bottom block**) represents a variety of workflow traces among the different possible graphs, 2D reconstructed images, simulations, topograms and 3D visualizations. The arrows indicate the order in which the visuals are normally analyzed by the experts/during the process analysis.

Electrical capacitance tomography data investigations can be split into a three-level workflow, as illustrated in Figure 3. Top left block (block 1, upper level) shows the basic relations between processes (1.1), measurement hardware, data capturing (1.2) and basic operations. This block illustrates hardware and software solutions dedicated to raw data acquisition output (1.3, 1.4) and image reconstruction module (1.5) coupled with sequences of output images (1.6) associated with the monitored process. Hardware part (including measurement units as well as sensors) is responsible for detecting the changes of the ongoing industrial process as well as the measurement data acquisition. Software part is associated mainly with the image reconstruction algorithms that can adopt various approaches to solve the inverse problem in terms of mathematical and engineering challenges. The resulting set of data (raw measurement data and reconstructed images) is the basis for further analysis and understanding the process while the further processes are illustrated in the next blocks.

Block 2 (upper right corner in Figure 3) presents basic visuals, which are often manipulated by domain experts upon starting the more in-depth data processing and analysis. Usually they start with basic plots of changes in raw data in time (2.1) as well as single or sequence of images (2.2, 2.3). They sometimes look for suitable forms of visualization, where the spatial distribution of flowing medium coupled with temporal changes in the process behavior is visible. During the pre-study we found out that one of the most favorable and useful views is the topogram (2.5), especially when the juxtaposition of data from different conditions of the process is analyzed (2.4). The choice of the form of data visualization depends on the type of the investigated process and on the particular information to be extracted from the data. However, the overall understanding of the process, especially tracking phenomena and both their causes and results within data for particular location and relevant time stamp is the most significant as well as challenging in terms of monitoring, diagnostics and control point of view.

As shown in block 3 in Figure 3 (bottom level), the most challenging part of working with data is interpreting and analyzing spatio-temporal datasets. Depending on the aim of the exploration, analysis modules can be ordered differently, yet still maintaining a logical workflow. Raw measurement data, reconstructed images, topograms and plots are all used as the input for this level. The ECT raw datasets provide insights about processes and can be eminently useful for understanding temporal changes inside an industrial installation. Image reconstruction is applied to prepare more efficient and intelligible representation of the data. The image reconstruction algorithms generate concentration maps of the spatial distribution of the material (for ECT permittivity), which are extremely useful for investigation of spatial changes occurring in the material distribution within the sensor.

The first step in the workflow of data analysis is the selection of an interesting time point, at which there are visible essential changes in the process. Usually it starts with an overview of concentration plots (Figure 3: 3.1). For the researchers and specialists in process analysis, a single look at the changes that occurred at the time of data measurement is enough to start working on data interpretation. Important changes in the process state are mapped into a spatial domain with the aid of the reconstructed images in 2D (Figure 3: 3.2,3.7) or 3D (Figure 3: 3.13,3.14). The level of homogeneity (analysis of the material distribution in space) allows for choosing an image area which significantly diverges from the expected results or provides new insights about the process. In this step, analysis is conducted using reference values from another part of the image. Comparison between different image areas, with color-scale modification, allows changes in the space domain to be presented in a more efficient way (Figure 3: 3.9). Data analysis workflow enforces understanding of changes in the time domain in the context of the spatial domain and vice-versa (Figure 3: 3.6,3.8,3.10–3.12). Sometimes, this job can also be supported by simulations and numerical calculations for parametric estimation of process properties (Figure 3: 3.3–3.5).

Typically, domain experts start data review with focus on features that are anticipated based on their knowledge supported by theoretical foundations and their experience. MATLAB-like or Octave-like software environments are supportive in this particular aspect of initial, low-level data exploration procedure. It is fairly straightforward to manipulate, process and visualize data with the use of command line tools. Experts are building scripts and sometimes even entire libraries or software packages dedicated to data processing to further expedite their work as they gain experience and proficiency [38].

### 2.3. Challenges in Analysis of Changes in Data in Space and Time

Previous work on spatio-temporal information visualization reveals a variety of approaches. There are multiple applications that emphasize proper visualization of the measurement data, especially in engineering context; from mechatronics to light and spectral distribution data [39,40]. The scope of proposed applications ranges from mapping of movable 3D visuals onto the localization maps through time, to spatio-temporal visualization methods, including decision making support as well as data intensive applications for emergency management important in handling disaster events [41,42]. Some works discuss challenges caused by simultaneous visualization of changes in both space and time domains. Overloading users with an excessively comprehensive representation (by for example adding a third spatial dimension) does not necessarily boost analysis, especially when considering temporal dimension concurrently. The authors showed an interesting concept of space and time cubes to extract development of localization distribution of phenomena over time, but they concluded that most of the times simpler 2D time visuals serve better [43]. It is somehow in line with our expectations about the specific role of combined 2D-spatial-temporal visuals that could be served in the case of ECT data analysis. One of the crucial insights from the pre-study was that some experts intrinsically and at the same time pervasively used topograms. As a matter of fact, topograms seem to be a rather simple type of visual form compared to the space and time cubes nonetheless at the same time give a broader, essential view. Topograms consist of a continuous series of single lines of pixels per single 2D cross-sectional reconstructed image. These lines are mapped onto a time series and stuck together to form a 2D topogram visual. Therefore, they convey rather time evolution changes mapped at the sensor point versus changes of localization spreading over time. However, this type of visual cue seems to satisfy requirements of the industrial process tomography data analysis. Domain experts adopted topograms as a sort of a reference point, a common denominator for process analysis workflow. Similar challenges and findings were presented for time-varying geospatial data study in [44], where authors describe results of comparison of existing visualization techniques in a task-based user study. Results showed that it is not possible to definitively generalize the human perception without differentiation between object shapes perceptiveness and changes conveyed by visuals. Aside from that, these large differences in user efficiency may arise due to different types of visualization illustrating the same data. To sum up, in the case of ECT data, the ongoing process is changing its properties and we are following these changes at the point of tomography sensor localization. For that reason these changes should be preferably followed at the point of tomography sensor localization as opposed to following localization and objects’ properties in time and space. In this context, term: "localization" is merely associated with sensing at some fixed point on the process route (i.e., flow loop). Yet, it could be cognitively perceived as travelling along the process phenomena with the flow along the time.

Hence, inspired by past research on spatial and temporal data analysis we aim to propose a new approach facilitating process tomography data analysis in industrial scenarios based on exploration of the visuals representing spatial and temporal features. The goal is to propose a new paradigm built around the interpenetrating, complementary spatio-temporal relations existing in the measurement data. The proposed new methodology has been evaluated using a prototype system based on a task-driven user study with participants being experts in industrial process tomography data analysis.

## 3. Interactive Spatio-Temporal Contextual Data Analysis

### 3.1. Spatio-Temporal Analytical Approach

The main goal of the proposed solution is to establish and maintain the central role of the spatio-temporal relationship inherent in the measurement data along the entire process of analysis as well as interpretation of the investigated phenomena. Hence, as we seek to establish this paradigm, we proposed an interactive timeline layer (ITL) as a mechanism for constant positioning of the rendered data around a common center of the analysis procedure regardless of the current focus of the user.

**Figure 4:**
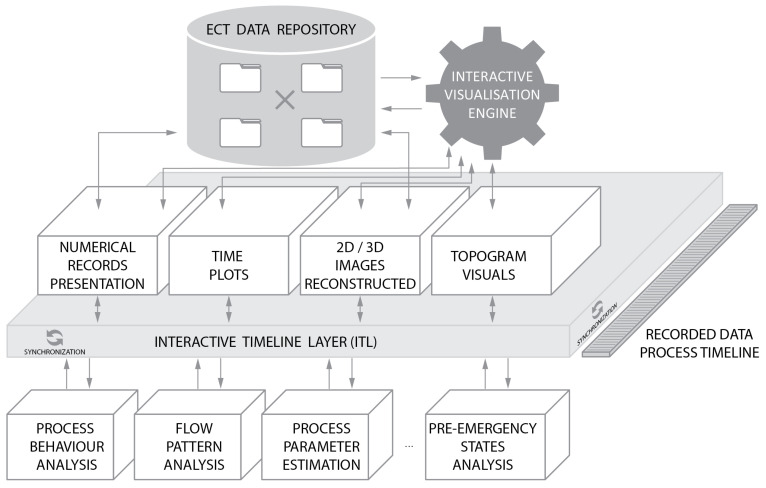
Schematic diagram of the Interactive timeline synchronization mechanism. Top layer serves as both: the ECT data repository as well as the engine serving requests for particular visuals. Visuals layer (second from the top) is bounded to the central Interactive Timeline Layer (ITL) that synchronizes the requested visuals with the corresponding time stamp of the process as recorded in the measurement data. Bottom layer represents particular purpose of the analysis that makes use of the ITL in accordance with the specific needs.

ITL contextually synchronizes the focal point of the analysis with the time stamp position on the actual recorded data process timeline. The context of synchronization is meant to couple the time position together with its nearest neighborhood, i.e., an additional, extra set of adjacent preceding and subsequent data. It is intended to be included within the pre-rendered chunk of data easily available to the user continuously throughout the entire workflow. Contextual neighborhood is composed of measurement data related to both space and time features representing sequence from a fixed length adjacent interval. Figure 4 illustrates the main idea of establishing the spatio-temporal interactive data presentation paradigm as a core module of the analysis system. The interactive timeline layer works as the interface between the upper data containers and the application cases depicted in the bottom of the picture. ITL takes care of synchronizing the multiple modality visuals such as plots or reconstructed tomograms with the actual process timeline regardless of the data chunk currently activated by the operator. The ITL interface allows the user to easily switch between analysis of the spatial features (inherent in 2/3/4D reconstructed images) and temporal sequence patterns (inherent in plots or topograms) preserving the context of the analysis.

The key asset of the proposed methodology is the systematic approach to data analysis procedure organized around the spatio-temporal data presentation regardless of the examined phenomena. The consistent arrangement of the data around the current focus chunk is a main advantage in comparison to the more chaotic data analysis loops employed by the experts for different analysis purposes as it can be observed in Figure 3, block 3. Another notable advantage is the possibility to adjust the focus and the adjacent neighborhood to the particular context of the data analysis. When looking at the general process understanding, a different, broader context resolution should be applied to data, in contrast to the investigation of the particular flow parameters. The opposite occurs when more spatial or temporal (depending on the case) details are required to draw important estimation insights

### 3.2. Prototype Design and Implementation

**Figure 5:**
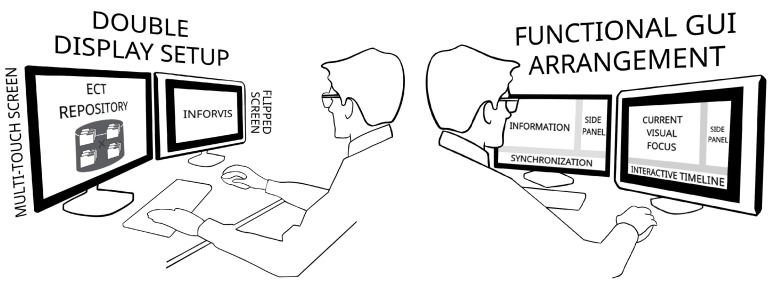
INFORVIS system functional prototype sketches. Left picture shows the double display set-up with a multi-touch interface capacity and a flipped screen deployed for ECT data analysis. Right picture presents functional GUI arrangement with the ITL timeline and information visuals screen implementation.

To experimentally verify the proposed methodology, the solution illustrated in Figure 4 has been implemented in a prototype data analysis system. The system serves as a complete workflow for interactive spatio-temporal data visualization and analysis (INFORVIS). The core functionality of the system is the interactive mechanism for switching the presentation mode following user actions, preserving the spatio-temporal context of analysis. Therefore, the ITL concept described in Section 3.1 plays the core role in enhancing abstraction of data manipulation and presentation relevant to the current user needs and in particular according to the specific context of analysis goal. Figure 5 shows functional sketch of the prototype system. One can see the essential arrangement of the double display set-up GUI presenting ECT repository data-driven interface. Graphical user interface (GUI) is split into three main areas: ITL visual timeline at the bottom, visual selection menu at right of the screen and the central visual display area covering majority of the screen from the top right.

**Figure 6:**
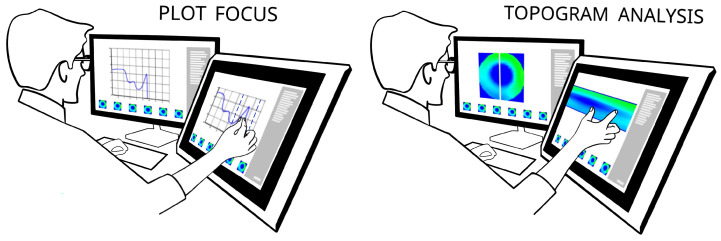
Prototype INFORVIS system use cases. Left picture: participant working with time plot selected on a far-right display and manipulated on a flipped multi-touch screen. Right picture: topogram analysis (far-right display) with a cut-plane indicated on a 2D reconstructed image (left, vertical display).

Figure 6 illustrates exemplary use cases: concentration plot interpretation and a topogram analysis. One can see a domain expert working on a data analysis using double display set-up with flipped screen and multi-touch interface. Prototype system supports simultaneous use of 2 displays. For example time plot selected by the user on a vertical screen is visible there (left screen) together with a detailed focus-view and manipulation being pursued on a flipped (right) screen as indicated on a left picture on Figure 6. System adjusts the bottom timeline strip display according to a current focus set by the user with a pinch-zoom two-finger gesture on a plot displayed on flipped screen. The right picture in Figure 6 shows user working with a topogram visual. Left display shows a 2D reconstructed image of a pipe cross-section with a vertical line representing the topogram plane and a bottom timeline showing the nearest neighborhood adjacent images. Correspondingly, right screen shows manipulation pursued by the user on a right screen.

**Figure 7:**
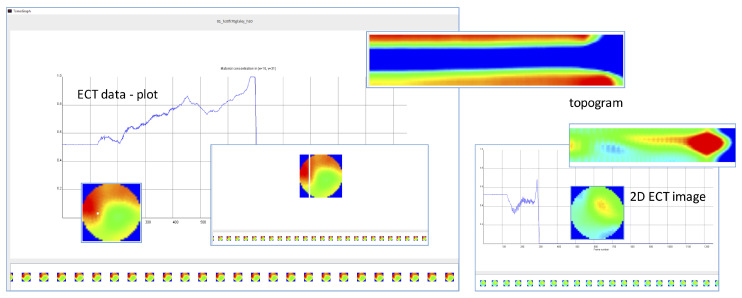
Screenshots of the INFORVIS system features. Demonstration of various interface elements and visual illustration implemented data workflow support features. From left: plot vs. time diagram, 2D cross-sectional single frame image, single pixel line time slice in the form of topogram. Bottom images illustrate consecutive time frames in the form of reconstructed 2D cross-sectional distribution images.

In addition to that, INFORVIS is designed to simplify and provide more user-intuitive manner of data handling and analysis. It supports various forms of data visualization in different arrangements, while facilitating proper navigation through both raw and processed data in compliance with baseline standards [2,15]. Figure 7 shows the selection of implemented graphical user interface elements corresponding to the visualization of 2D reconstructed images, average concentration data plots and topograms.

**Figure 8:**
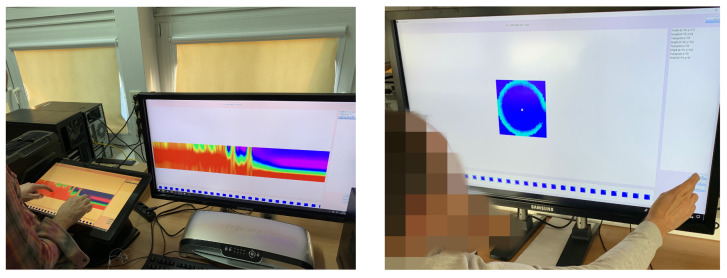
User working with the prototype system. (**Left**) two display setups (one horizontal multi-touch 20-inch surface plus one vertical classical 27-inch monitor). (**Right**) 40-inch vertical multi-touch display.

The prototype data analysis system was built to facilitate following the inherent measurement data relations during analysis. It was examined thoroughly using ECT measurement data captured for the gravitational silo flow of granular material, since this class of processes is characterized by dynamic phenomena occurring in the spatio-temporal domains. Initial design requirements were drawn from observation and semi-structured interviews with domain experts conducted after a shadowing study with baseline methods typically used in tomography measurement data analysis. Providing intuitive and comfortable mechanisms for browsing, managing and exploring the information in terms of continuous connections in time and space turned out to be a priority. To avoid introducing unknown interfaces, yet retain a tangible UI mode, a multi-touch surface was chosen. Multi-touch surface operation was implemented to avoid additional initial cognitive effort on top of the need for the user to learn the novel, proposed features for data manipulation. Therefore, intuitive gestures such as those used with smartphones and tablets, i.e., tapping, zooming, scrolling, pinching and panning were used. Such design and implementation minimized the need for additional navigation slidebars and icons, while preserving task-solving functionality. Figure 8 shows experts working with the system using single and dual screens.

The proposed design was tested successfully with small tablets, flipped and fixed medium size tabletop displays (20 inch), and full screen multi-touch 40 inch tabletop devices working under different principles of operation (equipped with infrared, optical and capacitance sensors). Synchronization between the devices and platforms is possible, enabling on-site investigation and collaborative exploration by remote expert users.

**Figure 9:**
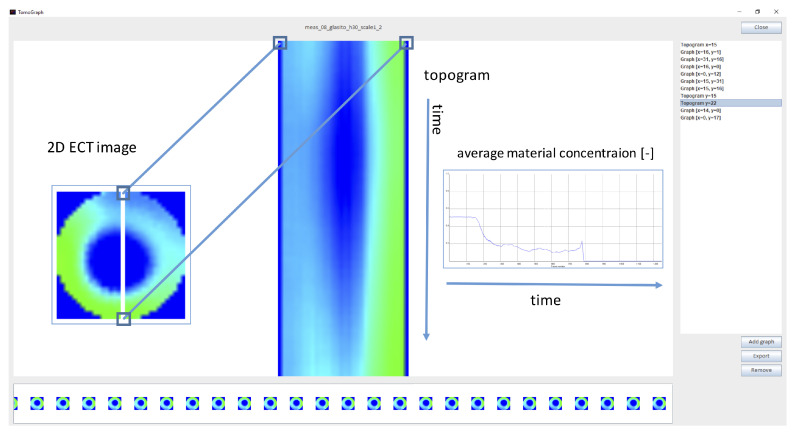
Illustration of how the system supports visualization and analysis transitionally, switching from 2D cross-sectional image space to a 2D temporal image (topogram) and back to the time plot diagram for a particular time point of choice.

The ultimate goal of ECT data analysis is to properly acquire measurements and interpret the process evolving inside the examined installation (the flow loop). Typically, the first choice of the operator during the analysis of flow parameters are the concentration profiles in the form of average plot diagrams or raw measurement records acquired from a single pair of electrodes. Later, the raw measurement data is transformed into images of material distribution inside the sensor space. These images allow further analysis thanks to generation of corresponding graphs and tomograms, which are employed to analyze various aspects of the investigated process (such as flow patterns and regimes, velocity and mass parameters assessment, etc.). A variety of visualization parameters can be helpful to domain experts. Some experts rely on average concentration visuals mainly for entire cross-sectional reconstructions, others generate profiles for single pixels located in characteristic regions of cross-sections and couple them with slice-based topograms. Both solutions are based on the temporal relations between these spatial elements of cross-sectional parameters. Figure 9 shows the simplified process for generating the temporal image (center) for a given slice of a cross-sectional plane (left) and then producing an average concentration plot corresponding to the selected slice over time (right).

The bottom stripe displaying consecutive 2D reconstructed images for subsequent measurement frames works as a contextual timeline control. It can easily be activated by a single tap on a chosen image. The contextual neighborhood is assigned a minimal fixed length in terms of total interval centered on the focal point of process timeline. It is possible to adjust the length of the neighborhood interval depending on the context of the analysis. Therefore, this is not only the implementation of the ITL concept but also an additional opportunity for further research on matching the appropriate neighborhood in a specific, proper context. The rest of the interface adjusts the current view to follow the timeline-indicated current frame, regardless of whether a magnified 2D image, topogram or plot is displayed at that particular moment in the main window above. The important feature is that setting the current focus is possible not only on the timeline bar but anywhere regardless of which of the visuals is analyzed. Transitioning between various visuals is possible by using the right-hand side panel, in order to ease tracing data features by switching smoothly between the visuals without losing the current time point on the way.

## 4. Evaluation Methodology

To evaluate the proposed methodology, a user study was conducted in controlled laboratory settings. First, participants followed data analysis scenarios to complete a set of given, specific tasks. During this stage, their actions were being recorded to investigate the workflow and performance. Results such as the task completion times were calculated based on the recorded actions. Additionally, some of the comments given by the experts during the study were extracted for further analysis. Secondly, immediately after the completing the assigned experimental scenario, each participant was asked to complete the NASA Task Load Index survey.

### 4.1. Research Questions

We aim at developing a methodology that enables analyzing and understanding the changes occurring in the measurement data in a more efficient manner. Our secondary goal is to enable effective browsing of the data, finding faster, process-relevant information in the data, and simultaneously minimize user’s effort. Hence, our research questions are as follows:

Q1. Would and how implementing the interactive timeline paradigm could improve the analytical procedures?
Q2. Does the implementation of up-to-date information display and control technologies influence the performance of the users?

Therefore, as we decided to implement prototype system, we formulated the following hypotheses:

**Hypothesis 1** **(H1).** *Deployment of the interactive timeline focus paradigm improves performance defined in terms of task completion time and accuracy*.

**Hypothesis 2** **(H2).** The ability to easily switch between forms of data presentation along the continuously synchronized spatio-temporal focus leads to the reduction of cognitive and workload indices.

**Hypothesis 3** **(H3).** Allowing users to employ multi-touch big screens and flipped displays coupled with a well-known set of control gestures gives users better comfort and improves their self-confidence when performing measurement data analysis.

To verify our research hypotheses we conducted a comparative, within-subjects user study using the baseline system and the proposed prototype system. We developed a study protocol and task-based study scenario specifically aimed at evaluating the H1 and H2 empirically through the set of quantitative and qualitative methods. Furthermore, we hope to draw conclusions on H3 based on mixed methods (such as shadowing, observation, interviews) employed throughout the experiments.

### 4.2. Deployment Conditions and Experimental Environment

The experimental evaluation was conducted within controlled laboratory settings of the Institute of Applied Computer Science, Lodz University of Technology (TUL). Two, computationally comparable, data analysis set-up arrangements were prepared to provide participants with the baseline and prototype systems access. Each system core was a modern PC station equipped with 32GB RAM memory, Intel i-7 7800 6-core CPU operating @ 3.5GHz and NVIDIA GeForce GTX1080Ti 11GB graphics operated under MS Windows 10 Pro x64 operating system, and was carefully installed and tested for seamless operation before employment.

The main difference between the systems was the data analysis software available on these two environments. Baseline system was equipped with GNU Octave and proprietary Matlab numerical calculation software packages as well as standard PC built-in applications and internet connected Google Apps (mainly Sheets, Drive and Docs). Especially Octave and Matlab provided specialized data manipulation capacity. Both were configured based on the pre-study brief survey with domain experts, who listed software and optional toolboxes that would help ECT measurement data analysis. One of the interesting examples of a very specific applications required by the experts and installed on the baseline system was the EIDORS package that is a research, open software extension to the Matlab package dedicated to EIT (Electrical Impedance Tomography) calculations and visualizations [38]. In Matlab-like (or Octave-like) software environment, one can follow all steps and verify understanding of data in process aspects at each consecutive time point. For the baseline comparison procedure, we equipped Octave and Matlab with a set of prepared scripts which allow the reading, plotting and visualization of data in a form of a single image sequence chosen from a particular selected range of data as well as topogram view. The participants can employ any script that can visualize data in a form which is the most appropriate to solve the particular task. Examples of such data visualization are presented in Block 1 and 2 in Figure 3 (upper level of the picture). In comparison, the proposed approach was based on the INFORVIS prototype application coupled with access to standard PC built-in applications and internet connected Google Apps (mainly Sheets, Drive and Docs). Both baseline and INFORVIS systems were equipped with two displays operated as normal computer screens as well as the ones supporting multi-touch navigation. One of those screens had ability to be flipped gradually from vertical to horizontal position smoothly.

To evaluate the proposed approach to ECT data analysis, a task-based study was proposed. The scenario was based on the analysis of ECT data which was interpreted in the context of the gravitational flow of bulk materials. This particular process was chosen due to the prevalence of bulk material flows in industrial environments [36].

### 4.3. Silo Flow and Measurement ECT Laboratory Set-up

Gravitational flow in silos is a typical process common in a variety of applications. It is an inherent constituent of both complex bulk solids systems and single containers used for the storage and protection of material. There is a wide variety of geometries, shapes and sizes available, depending on particular applications and material properties. The vast majority of solid materials in the industry is processed in the form of a granulate, most frequently transported with the use of pneumatic and gravitational flows. In the case of silo flow, the geometry of the tank, the properties of the granular material and the distance above the outlet determine various flow patterns, mainly regarding the mass and funnel [45]. The behavior of the material is also influenced by initial load density of granular material and the asperity of silo walls [46], along with various external and internal parameters, such as humidity, temperature etc., which could result in abnormal behavior, significantly different from the expected process line. Aberrant performance can result in possibly dangerous anomalies, related to the so-called “silo music”, which is caused by improper interactions between walls and particles in the silo. These effects can result in construction disasters, wherever mass flow is concerned [47]. Furthermore, in the case of tunnel flow, effects such as arching or ratholing can lead to breaks in the production process [45,48].

**Figure 10:**
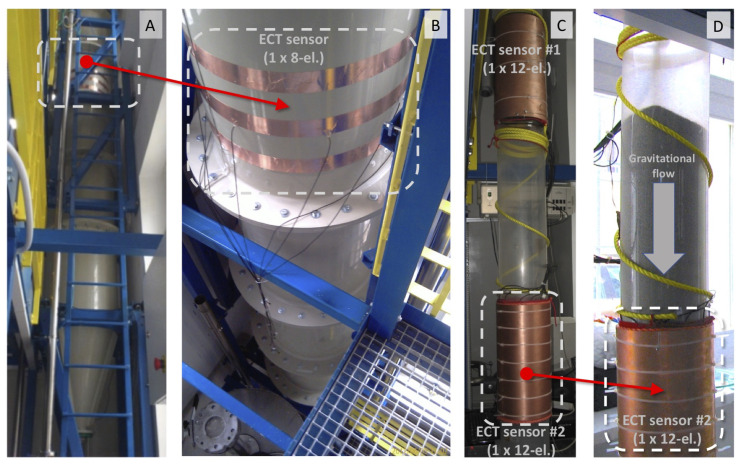
Slim silos at the Tom Dyakowski Process Tomography Lab in TUL. Left: pictures (**A**,**B**) show an industrial scale 7 m high silo for bulk solid processing equipped with a single plane, inner-mounted, ECT sensor. (**B**) shows a zoom-in view of ECT sensor shown in upper part of (**A**). Right: pictures (**C**,**D**) show a 2 m experimental silo model with two ECT sensors. (**D**) shows a zoom-in view of ECT sensor shown in lower part of (**C**). (**C**) reveals an empty silo model while (**D**) presents lower part of (**C**) captured during bulk solid flow.

Previous research was conducted at different facilities, with a range of silo sizes (full size, pilot, laboratory models) [34,36]. Theory and research practice show how to design relevant smaller scale models for experimental study that reflect real scale interactions within the flowing bulk as well as interactions between the bulk and the container [49]. Investigations regarding the flow of solid particles during silo unloading conducted using a variety of measurement techniques provided comprehensive knowledge about the process and the phenomena occurring during its progress. For this research, we have chosen the so-called slim silo type model (in which the relationship between the height and the diameter is equal to 10), as shown in Figure 10. ECT data was collected using a silo model of 2 m height and 0.2 m diameter.

Sand was chosen to imitate the granular material. A detailed description of the laboratory set-up can be found in [50]. The initial load density of sand was set at two different levels: loose and dense (i.e., compressed). The tests were conducted using both smooth and roughed-walled silos. The ECT sensor was installed at different heights relative to the silo outlet [50]. Experimental datasets were selected carefully to represent a range of possible phenomena recorded in both normal and extreme operational cases [51]. The input data consisted of raw measurement data and 2D reconstructed images gathered from a series of experiments monitored using a 12-electrode ECT tomography sensor located at different positions on the silo model. Each measurement frame contained 66 raw measurement records for distinct electrode pairs with no repetition. Image reconstruction was performed using a basic linear back projection (LBP) algorithm [3].

**Figure 11:**
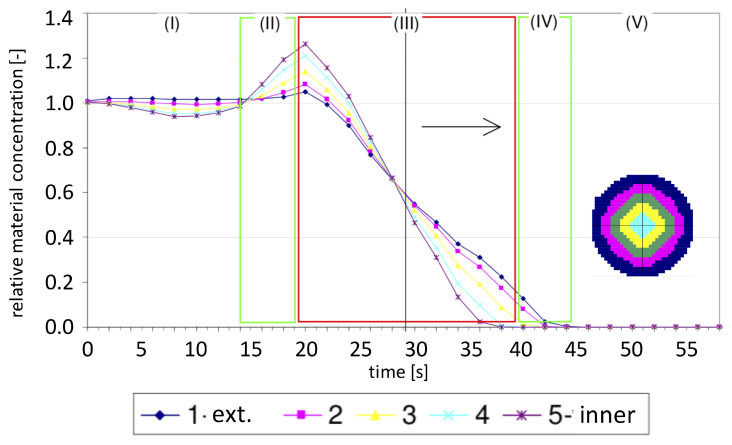
Diagram of ECT data changes over time during silo discharge. The five distinct linear plots show the average material concentration for five concentric rings from 1 (for the most external ring) to 5 (for the innermost circle). There are five stages in the process, indicated (**I**–**V**) in the upper part of the diagram.

Figure 11 shows a later stage of data processing, i.e., a plot of the average concentration in the silo discharging process. It reveals that the dynamics of the process are different for different zones of cross-sectional space. This plot illustrates how the gravitational flow process varies over time as it is divided into five stages (from I to V as indicated at the top of the picture in Figure 11).

Different flow parameters can be observed, analyzed and interpreted at different intervals. The phenomena that can be detected from the observations of the measurement data result from both the silo flow features as well as the ECT measurement modality properties. Interesting artifacts can be noticed within the intervals as well as on the boundaries (such as the border between stage II and stage III) [52]. Understanding the causes and effects of regularities and possible anomalies inherent in the ECT experimental measurement data is a key factor in successful interpretation of flow phenomena in the spatio-temporal domain.

### 4.4. Study Scenarios and Experimental Conditions

Evaluation of the proposed novel approach was based on an experiment in which experts were asked to solve a set of tasks, requiring them to conduct analysis and interpretation of ECT data related to gravitational silo flow. When trying to understand ECT data connected to the physical behavior of a process, it is usually necessary to often switch back and forth between temporal (raw data series, time plots, etc.) and spatial (reconstructed cross-sectional 2D images, volumetric 3D images) data visuals. The prepared scenarios included analysis of changes in measurement data important due to different flow process aspects. The experts analyzed silo flow behavior, determining symmetrical and non-symmetrical material distribution during silo discharge and the level of granular material vibrations and pulsations for different silo heights. The vibrations were analyzed in terms of their amplitude and frequency, and classified into three subsets (high-, mid- and low-amplitude vibrations) with relevant time intervals assigned. This procedure enabled the experts to determine the relations between the level of granulate and the vibration/pulsation parameters. During the study, the participants solved 10 tasks related to the nature of the funnel and mass flow. The ECT data were examined in both their spatial and temporal aspects. For the evaluation itself, 10 datasets of two types were prepared: 5 sets of measurement data for loose flow and 5 for dense flow. These two types of data refer to two types of flow conditions, since there were different artifacts included in the data for each of them. Generally, loose condition data may be more difficult to analyze, since some of the expected phenomena can be less evident, i.e., hard to spot in the data. Dense condition data can be characterized by easy to spot phenomena, such as a clearly visible boundary between the funnel and a stagnant zone, for example. The participants were asked to analyze different condition datasets in a randomized order, using two systems (the baseline and INFORVIS) to decrease the impact of bias.

### 4.5. User Study: Task-Based Evaluation

During the proposed data analysis tasks, different types of granular gravitational flow in the silo were investigated. ECT data were acquired during the gravitational flow of sand located in the silo model with 2 m height and 0.2 diameter. Possibility of initial packing of sand and wall roughness modification allows for generating four different flow conditions: (1) loose packing + smooth wall, (2) loose packing + rough wall, (3) dense packing + smooth wall and (4) dense packing + rough wall. The ECT sensor has been located at different heights above the outlet. Such arrangement of experiments allowed us to conduct the diagnostic of process behavior and described the relationship between flow and process condition.

The tasks were carefully designed to explore the spatial and temporal features inherently incorporated in the process measurement data. The experts were given brief instructions and presented with the sheets that included a short introductory text and 10 tasks, each composed of a description or question. Some questions were in single or multiple-choice form, while others had space for open answers or optional graphical elements in the form of an image grid to be sketched on by the participants. The scenario was designed in a manner to enforce detailed flow analysis going deeper into the process diagnostics gradually along the experiment. Additionally, there were several steps that allowed users for reflection about the way they execute tasks. Generally, the scenario is a complete set of complementary, correlated instructions and shall not be neither executed incomplete nor executed in a random order. This note is important to emphasize that we designed the entire experimental comparison between the baseline and proposed approach holistically. Although it is easier to explain their purposes in groups. Hence, tasks can be classified into three main clusters. The first set of tasks was designed to execute general, qualitative, decisive (judgement) instructions (T1, T10 and partially T5.). These were important to introduce participants into the working context of the study and allowed to assess users’ understanding of the analyzed process. These tasks allowed also for reflection on the differences between the baseline and proposed methods in terms of workflow and workload. The second group can be described as related mainly to performance. These required users to track down, indicate and describe specific phenomena (T2, T4, T5, T7 and partially T8). These tasks were designed to explore users performance based on their workflow. In addition to that, users could reflect on their usage of visuals and adjust their performance based on the technical requirements. The third type of tasks was related to assess, estimate, calculate and measure particular recorded flow effects (T3, T6, T8, T9, and partially T2). This group of instructions was aimed at measuring accuracy with respect to particular conditions and partially in relation to workflow performance.

In the first task (T1), participant analyzed data to indicate the flow type at a specific height (funnel/mass or mixed flow type). T2 was related to the investigation of pulsations localization. Additionally, the users were required to estimate basic parameters using empty cross-sectional mesh for sketching and note taking. They were encouraged to include numerical values, required phenomena and discovered effects. Next two tasks (T3, T4) explored the issue of symmetry analysis of granular material distribution (concentration) in the silo cross-section and its impact on occurring vibrations/pulsations. Both tasks required users to illustrate their conclusions with sketches and optionally numerical values. Following 2 tasks (T5, T6) were related to tracking the particular flow period in search of specific effects and describing them both in a qualitative and quantitative way. T7 was solely devoted to denoting the workflow sequence that helped participant to resolve the previous tasks. T8 was again focused on quantitatively estimating and mapping the analyzed phenomena in cross-sectional space. Furthermore, in the T8, participants were given an opportunity to make comments in a form of notes. Subsequently, T9 revised the estimated qualitative character of the examined flow coupled with quick quantitative estimation of predominant character over time. Task sequence concluded with T10 where participants were to indicate practical elements of the employed workflow that were most helpful on the way to solve T9.

**Figure 12:**
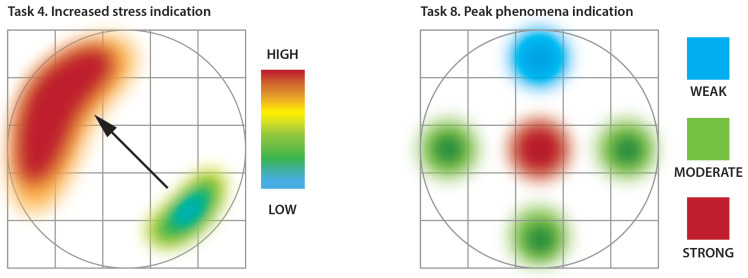
Heatmap illustrations for exemplary drawing tasks plotted on a background grid corresponding to the cross-sectional area of the measurement space. (**Left**) Stress indication, Task no.4 (dark red for maximum, blue for minimum. The arrow points toward the maximum values). (**Right**) Peak indication, Task no.8. (Dark red in the center for strong, light blue in top for weak, and green for moderate.)

To give the reader a deeper insight into the employed experimental scenario we present two representative tasks, namely T4 and T8, that require participants to both search for, estimate or calculate and finally indicate the resulting answers and map onto the space in form sketch. Figure 12 shows aggregated graphical answers for these two tasks that required the participants to sketch the patterns discovered in the data in the participants’ answer sheets. Task 4 (on the left in Figure 12) was related to flow symmetry analysis. It was expected to be one of the most demanding tasks, since possible differences in the material concentration values expected for this type of flow range in (2–5)% intervals at maximum. The participants were asked to sketch two regions: the one with the highest stress (corresponding to the greatest bulk concentration) and another with the lowest stress (corresponding to the smallest bulk concentration). Additionally, they were required to indicate the higher stress area using an arrowhead pointing from the lower stress area. All these drawings were designed to be sketched on a grid representing the cross-sectional area of the measured sensor space. The heat map shown on the left in Figure 12 shows the aggregated results for multiple correct answers, where dark red color shows the area of the highest stress, blue shows the lowest stress values and the arrow points from the lowest to the highest. Solving this kind of task required the participant to explore cross-sectional spatial data mainly, but at the same time keep track of time-adjacent frames in specific time windows.

The other task, T8 is shown on the right in Figure 12. Task 8 was designed to discover the peak phenomena that can be observed on a bulk concentration vs. time plot. The participants were required to explore the concentration values over time, in order to detect sudden peaks in values for different regions of a cross-section of the measurement space. This is another possible way of discovering flow asymmetry, including even slight differences in the measured values. Solving this type of task requires deep temporal analysis of different regions of the measurement space, including point-based (pixel) plots for different cross-sectional regions or multi-point (line of pixel-based) i.e., topogramic types of visualization, or even graphs based on total average concentration over time.

### 4.6. Participants, Procedure, Quantitative and Qualitative Assessment

The study consisted of n = 17 participants, each of whom were experts at the analysis of ECT measurement data. Results of 3 participants (out of 20 recruited initially) were excluded due to discontinuation of the scenario execution, participant drop-out and technical problems resulting in false timing results. Participants gave written consent and were relaxed before taking the experiments. Each of the participants was presented with the functionalities of both the baseline as well as the INFORVIS systems and given hands-on practice on an exemplary dataset. Subsequently, participants were shortly instructed about the experimental scenario and received a 5-page answer sheet. The experimental conditions were assigned almost symmetrically in a random way (9 out of 17 started with baseline and 8 out 17 started with INFORVIS). Even though the answer sheets were the same, i.e., independent of the condition, each participant was given only 1 answer sheet required for current condition just before starting the tasks. Entire study sessions were video and audio recorded for further analysis. Participants, answer sheets and recordings were coded and anonymized. Video and audio material was deleted after analysis.

As we sook to verify the hypotheses stated in Section 4.1 we applied a set of quantitative and qualitative measures. The baseline and INFORVIS prototype experimental conditions were inspected with the aid of standard parameters for the proposed task-based study, i.e., accuracy and task completion time. Additionally, we inspected the cognitive and workload index assessments and analyzed participants’ comments and remarks. Accuracy was assessed by comparing the answer sheets filled in by the participants against the carefully prepared ground truth answer tables. The tables were prepared beforehand by 2 different experts and double checked by yet another one (each of the three experts had more than 15 years experience). In case of the tasks that involved graphical responses or sketching, both the individual responses and the total cumulative response were obtained by interpolation aligning of the subsequent sketches and drawings with the grid. Scanned images were then aligned on distinct layers on top of the grid using a standard graphical software package. Decoded video material served as a mean of timing measure. Task completion times for entire experiments as well as for distinct tasks were decoded from the video afterwards all the experimental sessions were completed.

Upon finishing the tasks, the participants completed a survey concerning their overall user experience, their satisfaction with the system interface and the degree of challenge. In order to measure the effort required for task completion, a task load index was employed. The NASA Task Load Index (TLX) is a subjective workload self-assessment tool for estimating human workload parameters that has been developed in the 1980s by NASA for human factor-based evaluation of machine interfaces. There are six main categories of TLX self-evaluation: mental, physical and temporal demands, performance, effort and frustration. Further details on this method can be found in [53].

To analyze the rest of quantitative feedback we collected all the remarks noted by the participants in the answer sheets and encoded auditory records from the videos. After completing the scenario analysis, the users were also debriefed in a semi-structured interview.

## 5. Results and Discussion

### 5.1. Task Completion Time Results

**Figure 13:**
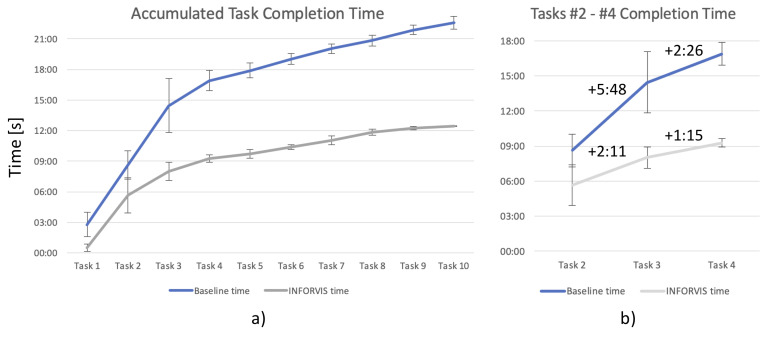
Task completion time diagrams for consecutive tasks throughout the study scenario. The upper dark blue line shows TCT with the baseline methodology. The lower grey line represents timings for the INFORVIS-based system. The vertical bars represent variation in the recorded timing results for all participants. (**a**) Accumulated TCT over the entire scenario for all the 10 tasks completed (left). (**b**) Segment of the three most demanding tasks: #2–#4 (right). The values presented above the segments of time plots indicate time increments for consecutive tasks.

The first quantitative results gathered for the experimental study were the time of completion (TCT). The TCT was measured for each of the tasks performed by the participants. The total aggregated TCT for completing all the tasks by each participant was also calculated by encoding the experimental videos. Figure 13 shows two plots of comparative curves of aggregated TCTs for the baseline and INFORVIS systems. Figure 13a) (on the left-hand side) shows accumulated TCT curves represented by the blue (upper) line for the baseline system and the grey (bottom) line for INFORVIS. For each task, there is an interval of variation shown as the standard deviation (SD) represented by a vertical bounded line. There is a significant difference in the TCT times for the baseline and INFORVIS systems, starting from the very beginning and increasing all the way towards the end of the experiment. However, the greatest difference is visible for the most demanding tasks, i.e., T2, T3 and T4. In order to better expose this part of the graph, a magnified fragment of it is presented on the right-hand side in Figure 13b). Values for completion time differences between consecutive tasks are highlighted above the plots. It can be concluded from the timing numbers that these tasks were the most challenging, at least in terms of execution time demands. Generally, the overall difference in the mean TCT shows significantly faster performance of the INFORVIS users when compared with the baseline system users.

**Figure 14:**
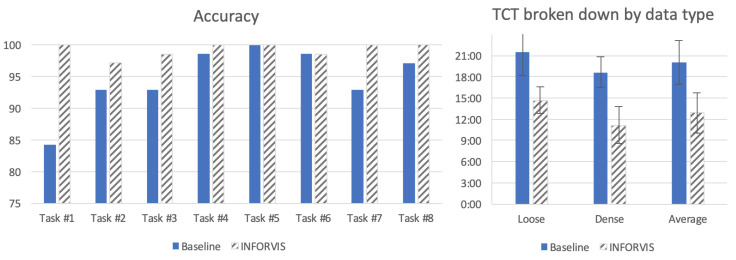
Accuracy bar plot and TCT broken down by conditions plot. (**Left**) average accuracy calculated for selected (quantitative) tasks. (**Right**) TCTs broken down by conditions and total average TCT. The first two pairs of bars represent two conditions, i.e., loose and dense, respectively. The last pair (furthest on the right) shows total average completion times for both conditions.

There is another TCT analysis presented on the bar diagram on the right-hand side of Figure 14. In order to determine the impact of different degrees of complexity inherent in the flow data on the obtained results, i.e., to examine different degrees of interpretation difficulty on the final results of the analysis, the TCT times were analyzed separately for loose and dense bulk solids flow data (corresponding to ’more difficult’ and ’easier’ to analyze, respectively). These TCT records were split into results for the two flow conditions of data and are presented in the bar chart given on the left of Figure 14. The results show a significant difference, as the data for the loose condition took more time (as expected) to process than that for the dense condition. Moreover, it is worth emphasizing that variance (illustrated by SD) remains at a similar level for both flow conditions. It can also be noted that with the INFORVIS system the TCT decreased substantially in comparison to the baseline set-up for both flow conditions; regardless of the level of difficulty.

### 5.2. Task Accuracy

The next and presumably the most important parameter we examined was the accuracy. On the left in Figure 14 the performance of the INFORVIS system is compared to that of the baseline in most of the tasks (note that not all the tasks designed within the study scenario aimed at accuracy assessment). Looking at tasks 2–4, one can see that not only is the TCT lower with the use of the INFORVIS system (refer to Figure 13), but there is also higher accuracy of answers (refer to Figure 14). Although some of the tasks show no difference (alternatively, the difference is not significant) between the conditions, there is not a single case of the baseline system providing better results than INFORVIS.

It is worth noting that the accuracy for the first three tasks was not perfect, especially in the case of the baseline set-up. In particular, correct answers were below 85% for Task #1 (multiple-choice question regarding the choice of flow type), while with the aid of INFORVIS there was 100% accuracy for this task. Observations of participants and careful analysis of the recorded videos revealed that they were more self-convinced about the correctness of the quick choices using baseline methods, so they did not double-check all the possible forms of data visualizations in order to come to a decision. Some of them later realized their error (after completing two, three or even four subsequent tasks) and corrected their answer, yet not all of them did so. This is one of the elements of data workflow where easy access to spatio-temporal dependent relations between the visuals can significantly help achieve much better interpretation. Participants were likely to use the topograms in addition to basic plots and the 2D cross-sectional images during analysis using the INFORVIS system, whereas when using baseline methods, the topograms were mostly omitted.

### 5.3. Cognitive and Workload Assessment

**Figure 15:**
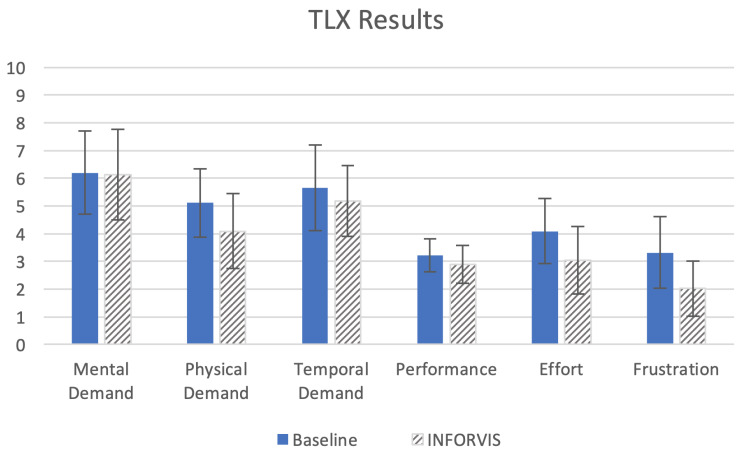
Task load index (TLX) results diagram for 6, self-assessed categories. Each category index is represented by a pair of bars: one for baseline condition (blue-color-filled, left-hand side bars) and another one for INFORVIS system condition (white-grey-hatched, right-hand side bars).

Another key aspect of the evaluation was the self-estimation of workload when completing the task with the aid of baseline methods vs. the proposed approach implemented in the INFORVIS prototype system. The results of NASA TLX show the overall slight superiority of the proposed INFORVIS system over the baseline set-up, as shown in Figure 15. Nevertheless, there was no significant difference in terms of mental demand, temporal demand and performance. Physical demand was estimated to be lower; the users also reported lower effort and less frustration when using INFORVIS.

Interestingly, the participants considerably underestimated their performance when using both systems. The average TLX scores for individual categories were also rather low compared to similar scores for any common web application or everyday interfaces. Interviews with participants revealed that the main reason for this was the perceived high difficulty of the entire analysis experimental scenario and low self-confidence caused by non-standard character of the required assignment. In fact, real, measured performance scores in terms of both the accuracy and completion times were far more than good (as can be seen in Figure 13 and Figure 14).

### 5.4. Additional Findings and Reflections

The participants were asked to indicate the order of the visuals they had used in the workflow as they completed the tasks. This question was asked twice in the survey: first after completing tasks #1-#4 and later at the end of the entire process. We observed induced auto-reflection on the part of the participants when filling-in these questions; some of them even started to review their previous answers. In particular, some of them started to invoke, browse and preview topograms (that previously had not been displayed by them when using baseline methods) to correct answers to previous tasks.

The participants were furthermore asked to assess the efficiency and the convenience of operating each type of visual. Graphs were rated as definitely the easiest to use and efficient form of visual depiction. Analysis using reconstructed images were satisfactory, while managing them was rated as inconvenient. On the other hand, the tomograms were described as convenient to use, although not sufficiently useful in terms of data exploration. Interestingly, the topograms were used far more often when working with INFORVIS and proved to speed up the TCT. They also enabled correct conclusions to be drawn from the data faster than traditionally, when topograms were mostly omitted.

Other exciting observations were made by shadowing the participants as they completed the tasks, as well as from video analysis and post-study interviews with the experts. Interestingly, four of the experts reported finding interesting (industrial process flow-related) features in the data that were not expected of them. Participants made comments indicating their research-related nature that was not possible to explain on the fly during the ongoing experiment. The answer sheets prepared for the study had no appropriate forms to note these unexpected findings. All the findings concerned unexpected patterns of pulsations and were related to the task of vibration analysis. All the cases had been found using INFORVIS prototype, when switching back and forth between the temporal concentration graphs generated by the participants for different locations in 2D cross-sectional space and the corresponding plots or reconstructed images. The sample is statistically insufficient to draw any definite conclusions, yet there were no similar findings reported for the baseline method. Moreover, two experts concluded it would have been almost impossible to reveal the same phenomena in the data using the standard baseline set-up. The usage of the multi-touch screens and the flipped-enabled display was also analyzed. Although we noticed a variety of approaches and different levels of expertise in productive use of multi-touch features of computer and application control there were no common conclusion that could be drawn to our best knowledge from this particular study. Moreover, flipped positioning of the screen was neither anticipated nor expected by the participants, hence no conclusions on that topic can be drawn from this research, either.

The study revealed that there is a need for more accessible and intuitive tools, which could significantly enhance the workflow of tomographic data analysis. Expert comments provided insights that it is not only accuracy that is required, but also a greater emphasis on enabling intuitive data exploration. As specialist teams are becoming more and more interdisciplinary, engaging non-technological experts, developing interfaces for data analysis becomes more important. Therefore, careful design with respect to the inherent spatio-temporal properties of the data corresponding to flow phenomena occurring in industrial installations could facilitate more efficient analysis and proper interpretation of data.

### 5.5. Summary of the Results

Research question Q1 (regarding displayed visuals vs data analysis performance) and related hypotheses H1 (employing ITL mechanism for improving performance) and H2 (ease of time-space data manipulation for reduction of operator workload) have been successfully verified and positively confirmed. Results of scenario-based experiments related to task completion times (TCT results in Section 5.1), demonstrated substantial improvement in analytical tasks execution timing for both entire scenarios as well as for distinct tasks. Hypothesis H1 also invoked success rates and accuracy measures to be compared with the baseline system additionally to TCT measurement. Accuracy results (Section 5.2 showed improved accuracy for most of tasks, with an average of approx. 5% superiority, while some of them the difference was substantial (approx. 15% better performance in favor of the proposed approach). H2 referred to an assumption of additionally reduced cognitive and physical workload when using the proposed approach implemented in the prototype INFORVIS system. NASA TLX results presented in Section 5.3 partially confirmed that assumption. Participants reported lower physical demand, lower cognitive effort and less frustration when using the prototype system in comparison with the baseline. On the hand mental as well as temporal demands were assessed at the same level in two conditions. Interestingly, experts estimated their performance almost equally (the average difference in favor of INVORVIS prototype was inconclusively small) while actual recorded results (both TCT and accuracy) for most tasks were improved. Secondly, the assumptions made about the of the influence of the employment of specific display technologies and multi-touch interfaces in the performance of the users formulated as research question Q2 and specified in hypotheses H3 have not been confirmed. We observed positive impact of these technologies and the interactive set-up on the workflow and performance of some of the participants. Certain participants verbally expressed their satisfaction with the possibility to use multi-touch surfaces and well-known gestures, though we have not been able to draw any definite conclusions in this matter based on the user study. We have not been able to decode any significant correlation between the usage of these elements and neither performance nor the task workload index results from the recorded experimental videos. Therefore, we must recapitulate the results on Q2 and H3 as inconclusive at this stage of research.

The implementation of the results of this research will be especially supportive to the experts working in the field of process and chemical engineering as well as the designers that develop industrial monitoring and control systems. The implementation of innovative solutions in this area requires the development of data processing algorithms along with the entire data structures related to the monitored process; no matter if the eventual system is based on process tomography sensing or not. As the tomography data processing may lead to a better understanding of the industrial process behavior it will be beneficial also to the designers of the flow facilities.

### 5.6. Potential for Further Research-Future Work

The proposed methodology was demonstrated using ECT measurement data of gravitational bulk solids flow. It would be interesting to further evaluate the proposed features of instantaneous switching between the space and time domains using different visuals on other dynamic process tomography measurement data [5]. We plan to conduct such a study using industrial pneumatic conveying of powders processes, especially that previous work has shown that it is difficult to automatically process ECT data of phenomena occurring in this class of flows [54,55]. Investigation of pre-emergency states and patterns in the monitored flows could be especially relevant. The motivation behind the future study seems preeminent, as its goals could include foreseeing and preventing unwanted events and flow rig failures [5,25]. Advances in the analysis of big data experimental datasets open new possibilities for using the tool [37]. New capabilities in the fields of data mining and pattern recognition could contribute to better understanding and increased control over the investigated process. Web-based implementation of the solution would facilitate multi-platform operation, as well as broaden the potential for data exploration through crowdsourcing [56,57]. Moreover, as reported in Section 5.4 there exists potential for systematic investigation of rare phenomena occurring during industrial flows as well as in other areas such as temperature field control [54,58,59]. We noticed that experts employed different yet, convergent patterns of workflow in the course of analysis of the experimental scenario datasets. It seems that this area deserves more attention. Especially since our approach enables composing different data analysis workflows for specific/different processes in a flexible way and could act as a means to an end/to potential findings. Future research should focus on how to combine expert analysis workflows with use of eye tracking methods [60], possibly combined with motion tracking techniques [61] in order to obtain deeper understanding of how the visual artifacts could be optimized further. This approach could be beneficial, especially for further exploration of the unexpected information found by some participants in the data during pulsation analysis, as discussed in Section 6 of this paper. Although EEG interfaces are still not mature enough to be fully effective in professional contexts, they could be used to research neurocognitive workload reduction [62,63,64]. Our tests are limited to a laboratory setting, yet there is not much difference between real conditions and our study scenario. However, we are aware that there is a huge gap between the kind of offline tomography data exploration we focused on in this research, and the on-the-go, on-the-fly, ongoing industrial flow measurement data analysis for decision making purposes. We aim to explore such intensive conditions in a separate, closer to an ’in-the-wild’, study. Although we found no limitations in terms of data scalability within this study, more tests involving huge amount of data will surely set the boundaries for applicability in terms of prototype implementations.

Although we tried to design the prototype system and study the proposed concepts with appropriate care, we recognize that our approach is prone to limitations. The most important issue is we designed for a specific group of expert users. Although the system being tailored to the needs of the experts is not a limitation itself, the obtained insights are also limited to a user group of experts. Beginner and advanced users may have significantly different preferences for system design, especially in terms of user interface feel and look. Consequently, further work is required to explore the proposed interactive timeline ideas for less proficient users. Finally, while we found the results of this research to be inconclusive with regards to research question Q2 and hypothesis H3 (both related to deployment of modern technologies such as tangible interfaces, flipped, multi-touch surfaces, etc.) this area deserves deeper exploration in terms of novel multimodal interaction techniques pervasively penetrating our daily life [65].

## 6. Conclusions

This work shows a novel approach towards interactive spatio-temporal, process tomography measurement data analysis. The proposed interactive timeline concept is demonstrated using a prototype system for electrical capacitance tomography data and has been evaluated with a task-based user study. Experiments were conducted on gravitational bulk solids flow ECT datasets of diverse levels of interpretation difficulty. Quantitative results showed that the proposed approach assisted experts by automatically providing required visuals (plots, 2D reconstructed images and topograms) that lead to improved accuracy, reduced task completion times and decreased workload. Moreover, a user study suggested that the convenience of switching between the space and time domains in different arrangements enabled more detailed and accurate interpretation. The proposed approach outperforms the traditional method of data exploration and interpretation, no matter the level of industrial process measurement data difficulty, as demonstrated for two conditions for gravitational flow in silos (loose and dense packing density). The principles of operation for the novel measurement data analysis tool can be transferred to other types of measurement modalities, both for process tomography and other classes, especially for incomplete or insufficient data or whenever any data uncertainty is present. There is great potential for employing the proposed solution to other industrial measurement data captured from different processes. The most interesting application would be to employ this type of analysis as a first step on the way to automating data processing tasks that are currently impossible to automate. Preparing classes of annotated data for crowd sourcing and machine learning systems could be an interesting example.

## Abbreviations

The following abbreviations are used in this manuscript:

ECT	Electrical Capacitance Tomography
EEG	Electroencephalography
EIDORS	Electrical Impedance Tomography and Diffuse Optical Tomography Reconstruction Software
EIT	Electrical Impedance Tomography
INFORVIS	Interactive System for Spatio-temporal Data Visualization and Analysis
ITL	Interactive Timeline Layer
LBP	Linear Back Projection
NASA TLX	NASA Task Load Index
TCT	Task Completion Time
TUL	Lodz University of Technology
SD	Standard Deviation
2D/3D	Two/Three Dimensional
